# Engineering osteoporosis-related bone organoids: a mechanism–module–functional readout framework for disease modelling and biomarker translation

**DOI:** 10.3389/fcell.2026.1938041

**Published:** 2026-09-03

**Authors:** Xiaoping Lv, Xiaojuan Ran, Changmei Wei, Xuexin Liu, Jianxia Wang, Lijing Huo

**Affiliations:** 1 Hebei Medical University, Shijiazhuang, Hebei, China; 2 Department of Clinical Laboratory, Hebei General Hospital, Shijiazhuang, Hebei, China; 3 Beijing MDTK Biotechnology Ltd., Beijing, China

**Keywords:** biomarker translation, bone organoids, mechanical signaling, osteoimmunology, osteoporosis, vascular microenvironment

## Abstract

Osteoporosis is a systemic skeletal disorder characterised by reduced bone mass, microarchitectural deterioration and increased fracture risk. Its pathogenesis extends beyond an imbalance between bone formation and resorption and involves osteoimmune dysregulation, vascular insufficiency, oxidative and inflammatory stress, and altered mechanical signalling within the bone microenvironment. Conventional two-dimensional cultures cannot adequately reproduce the three-dimensional matrix organisation, spatial multicellular interactions and mechanical properties of bone tissue, whereas animal models are limited by species-specific differences in bone remodelling, immune regulation and disease progression. Bone organoids integrating human stem/progenitor cells, bone-resident cells, immune and endothelial components, biomimetic matrices and dynamic culture systems provide a promising intermediate platform for modelling osteoporosis-related microenvironmental dysfunction. Unlike previous reviews that broadly summarised bone organoid construction and applications across skeletal diseases, this review focuses specifically on osteoporosis and proposes a mechanism–module–functional readout framework. This framework translates osteogenic suppression, RANKL/RANK/OPG-mediated osteoclast activation, immune–inflammatory amplification, vascular impairment and mechanical unloading into selectable organoid modules with measurable endpoints, including matrix formation, mineralisation, osteoclast resorption, inflammatory and oxidative-stress responses, vascular network function and mechanosensitive signalling. We further discuss how these models could connect candidate molecular signals with human-relevant three-dimensional functional phenotypes, thereby supporting biomarker discovery, drug-response assessment and patient stratification. However, organoid-derived candidates should not be regarded as clinically validated biomarkers without confirmation in independent patient cohorts and prospective clinical studies. Standardised construction, reproducible quality control, functional maturation and clinically anchored validation will therefore be essential for advancing osteoporosis-related bone organoids from experimental disease models towards biomarker translation and precision medicine.

## 1 Introduction

Osteoporosis is a systemic metabolic skeletal disorder characterized by reduced bone mass, deterioration of bone microarchitecture and an increased risk of fragility fractures. With population aging, osteoporosis-related fractures are contributing increasingly to disability, loss of mobility, long-term care needs and socioeconomic burden. Elucidating the mechanisms underlying osteoporosis and establishing experimental models suitable for mechanistic investigation, drug evaluation and biomarker translation therefore remain major research priorities (Clynes et al., 2020; Shen et al., 2022).

Osteoporosis has traditionally been attributed to an imbalance in bone remodeling caused by impaired osteoblast function and enhanced osteoclast activity. However, increasing evidence indicates that its pathogenesis extends beyond abnormalities in individual bone cell lineages and involves endocrine alterations, osteoimmune dysregulation, chronic inflammation, oxidative stress, vascular dysfunction, extracellular matrix changes and insufficient mechanical stimulation (Zhao et al., 2025). These processes interact within the bone microenvironment and collectively shift homeostasis toward a proinflammatory, proresorptive, and antiosteogenic state. Osteoporosis research must therefore address multicellular and microenvironmental interactions rather than isolated pathways alone.

Current osteoporosis research relies predominantly on two-dimensional cultures and animal models. Two-dimensional systems permit controlled investigation of individual variables but do not adequately reproduce the mineralized extracellular matrix, spatial cellular organization, multicellular interactions, or mechanical environment of bone. Animal models can reproduce systemic bone loss, microarchitectural deterioration, and reduced bone strength and therefore remain essential for preclinical research. Nevertheless, species-specific differences in bone turnover, immune regulation, endocrine responses, and disease progression limit their ability to reproduce the human osteoporotic microenvironment. Dynamic interactions among human bone, immune, and vascular cells are also difficult to examine directly *in vivo*.

Osteoporosis-related three-dimensional bone models may help fill this experimental gap. By integrating human stem or progenitor cells, bone-associated cells, immune and endothelial components, biomimetic matrices, bioprinting, microfluidic perfusion, and dynamic culture, these models can reproduce selected features of matrix formation, mineralization, formation–resorption coupling, and disease-related microenvironmental dysfunction (Nicolas et al., 2020). They also permit controlled cellular composition, spatial organization, and longitudinal assessment of human cell interactions. However, these models should be considered complementary intermediate platforms rather than substitutes for animal studies or clinical investigation. In this review, bone organoids refer primarily to self-organizing constructs, whereas scaffold-based, bioprinted, and microfluidic systems are described as engineered organoid-like bone models or bone-on-a-chip systems. Collectively, they are termed osteoporosis-related three-dimensional bone models.

Recent reviews have summarized bone-organoid construction, standardization, and emerging engineering technologies (Lou et al., 2025), compared preclinical osteoporosis models across *in vivo*, *in vitro*, three-dimensional, organ-on-a-chip, and computational approaches (Plank et al., 2025), and specifically examined bone organoids for osteoporosis modeling and translation (Liu et al., 2026). These studies define the biological and technological landscape of the field. However, a fit-for-purpose map linking individual osteoporosis mechanisms to the minimum required cells, matrix properties, spatial and temporal organization, controlled inputs, functional endpoints, matched controls, evidence maturity, and failure criteria remains underdeveloped.

Accordingly, this review is organized around a mechanism–module–functional readout framework rather than construction technology alone. The framework is intended as application-dependent design guidance, not as a validated universal protocol. It identifies when a three-dimensional platform provides information beyond conventional two-dimensional culture, distinguishes experimentally demonstrated capabilities from prospective features, and connects platform selection and quality control with functional validation and biomarker evidence grading.

## 2 Mechanistic networks of osteoporosis and their translation into model requirements

**FIGURE 1:**
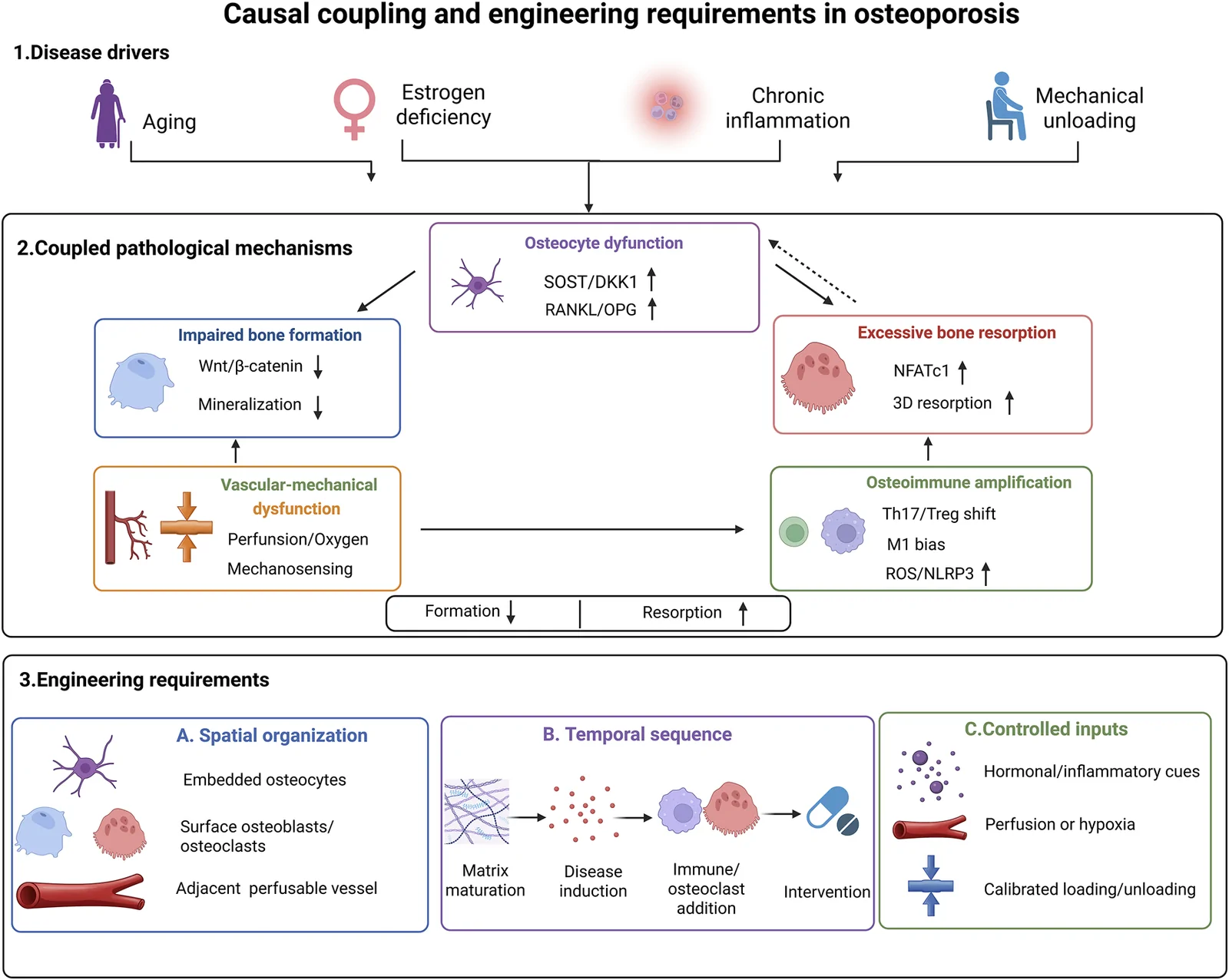
Causal coupling and engineering requirements in osteoporosis. Aging, estrogen deficiency, chronic inflammation, and mechanical unloading converge on osteocyte dysfunction. Increased SOST/DKK1 suppresses Wnt/β-catenin signaling and mineralization, whereas an elevated RANKL/OPG ratio promotes NFATc1-dependent osteoclastogenesis and matrix resorption. Osteoimmune amplification, characterized by a Th17/Treg shift, M1-skewed macrophage activation, and increased ROS/NLRP3 signaling, cooperates with reduced perfusion, oxygen availability, and mechanosensing to reinforce formation–resorption uncoupling. Engineering these mechanisms in three-dimensional bone models requires matrix-embedded osteocytes, surface-accessible osteoblasts and osteoclasts, adjacent perfusable vasculature, staged matrix maturation and disease induction, and controlled hormonal, inflammatory, vascular, and mechanical inputs. Solid arrows indicate primary causal relationships, whereas the dashed arrow denotes feedback from excessive resorption to osteocyte dysfunction. Created with BioRender.com. Publication license number: TJ2A50XFGI.

Osteoporosis reflects a network-level failure of bone homeostasis rather than isolated changes in osteoblasts or osteoclasts. Within the bone multicellular unit, osteocytes coordinate formation-resorption coupling, while immune, vascular, matrix, and mechanical signals regulate remodeling (Song et al., 2022). Aging, estrogen deficiency, inflammation, and mechanical unloading converge on the Wnt/sclerostin and RANKL/RANK/OPG axes, oxidative-inflammatory pathways, vascular-osteogenic coupling, and mechanosensitive signaling. Their interactions shift the bone microenvironment toward reduced formation, excessive resorption, and impaired repair (Figure 1).

Osteoporosis-related three-dimensional bone models need not reproduce this entire network. Instead, each model should capture the minimum causal pathway required for its intended use. The following subsections define the essential disease feature, cellular and matrix components, spatial and temporal organization, functional endpoints, controls, and failure criteria for each mechanism. When universal quantitative thresholds are unavailable, disease relevance should be assessed using predefined effect sizes relative to healthy, disease, and mechanism-specific intervention controls.

### 2.1 Osteocyte-centered disruption of bone formation–resorption coupling and the RANKL/RANK/OPG axis

Bone remodeling depends on the coordinated coupling of osteoblast-mediated bone formation and osteoclast-mediated bone resorption. Canonical Wnt/β-catenin signaling is a major regulator of osteoblast differentiation, matrix production, and mineralization (Song et al., 2025). Sclerostin and DKK1 inhibit this pathway: sclerostin binds to LRP5/6 and antagonizes canonical Wnt signaling, whereas DKK1-mediated Wnt inhibition reduces bone formation and produces an osteopenic phenotype (Li et al., 2005; Li et al., 2006). Increased activity of these Wnt antagonists in osteoporosis-related conditions can therefore impair osteoblast differentiation, osteoid deposition, and mineralization.

Enhanced osteoclastogenesis constitutes the corresponding resorptive component of this imbalance. Osteoclast differentiation from monocyte-macrophage-lineage precursors is primarily regulated by macrophage colony-stimulating factor and receptor activator of nuclear factor-κB ligand (RANKL). Binding of RANKL to RANK activates TRAF6-dependent NF-κB, MAPK, and NFATc1 signaling, thereby promoting osteoclast differentiation, maturation, and resorptive activity. Osteoprotegerin (OPG) acts as a soluble decoy receptor that limits the RANKL-RANK interaction. An increased RANKL/OPG ratio therefore favors excessive osteoclastogenesis and resorption-dominant remodeling. Osteocyte-derived RANKL has also been shown to contribute directly to increased osteoclast formation and cancellous bone loss following estrogen deficiency (Fujiwara et al., 2016). Osteocyte dysfunction can therefore suppress bone formation while promoting bone resorption.

Formation-resorption coupling is bidirectional and time-dependent. Experimental studies have shown that sclerostin can increase osteocytic support for osteoclastogenesis by increasing the RANKL/OPG ratio. Conversely, osteoclast-derived signals can promote the transition from resorption to formation. For example, osteoclast-derived leukemia inhibitory factor suppresses sclerostin expression in osteocytes and enhances Wnt/β-catenin-dependent bone formation (Koide et al., 2017). These reciprocal interactions suggest that osteoporosis may involve not only excessive resorption or impaired osteoblast differentiation, but also inadequate bone formation after resorption. Formation and resorption should therefore be evaluated as a coordinated sequence rather than as independent endpoints.

This distinction has direct implications for model design. Two-dimensional culture remains appropriate for examining isolated Wnt or RANKL signaling and lineage-specific differentiation. A three-dimensional platform is justified when the objective is to reproduce matrix-embedded osteocyte signaling, surface-localized osteoclast resorption, matrix-derived coupling cues, or the transition from resorption to formation. Such models should incorporate osteogenic and osteocyte-like cells within a mineralized, resorbable matrix while providing osteoclast precursors with controlled access to the matrix surface. Matrix maturation, disease induction, and osteoclast addition should occur at defined stages. Validation should integrate Wnt/β-catenin activity, SOST and DKK1 expression, the RANKL/OPG ratio (Wijenayaka et al., 2011), matrix formation and mineralization, osteoclast polarization, and quantitative three-dimensional resorption. A valid osteoporosis phenotype should maintain cell viability and structural integrity, produce a reproducible functional imbalance, and show at least partial reversal after mechanism-specific intervention. Molecular changes without corresponding alterations in matrix formation or resorption are insufficient to demonstrate disrupted remodeling coupling.

### 2.2 Imbalance in immune cells and the regulation of bone metabolism

The bone marrow is both a hematopoietic niche and an osteoimmune microenvironment in which immune cells interact with bone-associated cells to regulate bone homeostasis. In osteoporosis, immune regulation can shift toward proinflammatory and osteoclastogenic activity, thereby increasing bone resorption and suppressing bone formation.

T cells are key regulators of osteoimmune crosstalk (Okamoto and Takayanagi, 2023). Th17 cells promote inflammation and osteoclastogenesis by secreting IL-17, IL-6 and TNF-α, increasing.

RANKL expression and enhancing osteoclast differentiation (Qi et al., 2025). In contrast, regulatory T cells (Treg cells) inhibit osteoclastogenesis through IL-10-, TGF-β-, and CTLA-4-mediated mechanisms. In postmenopausal osteoporosis and inflammatory bone loss, the Th17/Treg balance has been reported toward Th17 dominance, resulting in increased inflammatory cytokine production, enhanced RANKL signaling and accelerated bone loss (Lu et al., 2025).

B cells also regulate bone remodeling through context-dependent effects. Under homeostatic conditions, they can produce OPG and help limit osteoclastogenesis. Under estrogen-deficient, inflammatory, or otherwise dysregulated immune conditions, B cells can express RANKL and promote osteoclast formation. Estrogen deficiency may therefore disrupt the B-cell-derived RANKL/OPG balance and further enhance osteoclast activation (Parolini, 2025; Yao et al., 2025).

Macrophages are another important component of the osteoimmune microenvironment (Yan et al., 2024). Proinflammatory M1-like macrophages release TNF-α, IL-1β, and IL-6, promoting osteoclast differentiation and inhibiting osteoblast function. In contrast, reparative M2-like macrophages produce IL-10 and TGF-β and contribute to inflammation resolution and bone repair. Aging, estrogen deficiency, and inflammatory stimulation can favor M1-like activity and amplify inflammatory bone resorption (Wang W. et al., 2022). However, macrophage activation exists along a functional continuum; therefore, the M1/M2 classification should be treated as an operational framework rather than a strict binary distinction.

These immune-cell populations do not act independently. Th17- and macrophage-derived cytokines can increase RANKL expression and osteoclast precursor sensitivity, whereas B-cell-derived RANKL and OPG further modify the same regulatory axis. Together, Th17/Treg imbalance, altered B-cell-derived RANKL/OPG regulation, and proinflammatory macrophage activation form an interconnected network that promotes bone resorption and suppresses bone formation (Yang et al., 2024).

These context-dependent interactions have direct implications for model design. Two-dimensional culture or conditioned-medium experiments remain appropriate for testing the direct effects of a defined cytokine or immune-cell product. A three-dimensional platform is justified when the objective is to reproduce immune-cell migration, contact-dependent signaling, local cytokine gradients, matrix retention of inflammatory mediators, or the spatial coupling of immune activation to bone formation and resorption. Depending on the research question, defined T-cell, B-cell, or macrophage populations should be introduced into a bone-forming construct before disease induction, during inflammatory activation, or after matrix maturation. The timing and route of immune-cell incorporation should be reported explicitly.

Validation should combine immune-state measurements with bone-functional endpoints. Relevant readouts include the Th17/Treg balance, B-cell RANKL/OPG expression, macrophage phenotype and secretory profile, inflammatory cytokine production, osteoclast differentiation and resorption, and osteoblast-mediated matrix formation and mineralization. Controls should include bone constructs without immune cells, unstimulated immune-cell controls, defined cytokine-stimulation controls, and mechanism-specific interventions. Because no universal Th17/Treg or M1/M2 threshold applies across donors and culture systems, disease relevance should be assessed using predefined changes relative to matched controls. Increased cytokine production or altered immune-cell proportions without a reproducible change in bone formation or resorption are insufficient to demonstrate osteoporosis-related osteoimmune dysfunction.

### 2.3 Inflammatory cytokines, oxidative stress and NLRP3 inflammasome activation

Inflammation and oxidative stress form a reinforcing network that promotes bone resorption and suppresses bone formation. Proinflammatory cytokines, including TNF-α, IL-1β, IL-6, and IL-17, increase the sensitivity of osteoclast precursors to RANKL and enhance osteoclast differentiation and survival. The same mediators can inhibit osteoblast differentiation, matrix production, and mineralization. TNF-α acts synergistically with RANKL through NF-κB and MAPK signaling, whereas IL-1β supports osteoclast survival and resorptive activity. IL-6 and IL-17 further reinforce inflammatory signaling and RANKL expression.

Reactive oxygen species (ROS) function as physiological signaling molecules during bone remodeling, but sustained ROS accumulation promotes osteoporotic bone loss (Marques-Carvalho et al., 2023). Excess ROS activates NF-κB and MAPK signaling, enhances osteoclastogenesis, and impairs osteoblast survival and differentiation. Inflammatory cytokines can increase mitochondrial ROS production, while ROS can amplify cytokine signaling, creating a self-reinforcing inflammatory-oxidative loop. NF-κB-dependent priming increases the expression of NLRP3 and pro-IL-1β, whereas mitochondrial ROS and damage-associated molecular patterns can promote inflammasome activation. Activated NLRP3 recruits caspase-1, leading to the maturation of IL-1β and IL-18 and further amplification of inflammatory bone resorption (An et al., 2019). Bone matrix degradation products can also activate NLRP3 and promote osteoclast differentiation, suggesting that resorption itself may reinforce local inflammation (Alippe et al., 2017).

The biological significance of this network depends on the magnitude, duration, and spatial distribution of the inflammatory stimulus. Transient ROS generation participates in normal cell signaling, whereas sustained oxidative stress is more likely to impair bone homeostasis. Similarly, short-term exposure to a high cytokine concentration may cause nonspecific injury rather than reproduce chronic osteoporosis-related inflammation. In three-dimensional constructs, diffusion limitations and central hypoxia can independently increase ROS and inflammatory signaling. These culture-related effects must be distinguished from the intended disease mechanism.

These considerations have direct implications for model design. Two-dimensional culture remains suitable for testing isolated cytokine, ROS, or NLRP3 pathways. A three-dimensional platform is justified when the objective is to reproduce cytokine and oxygen gradients, matrix retention of inflammatory mediators, multicellular feedback, or the spatial relationship between inflammation and bone remodeling. Disease induction should begin after the construct has established a stable baseline phenotype, and cytokine or oxidative stimulation should be applied within a predefined dose and time window. The induction protocol should alter inflammatory and bone-remodeling functions without causing generalized loss of viability.

Validation should integrate inflammatory, oxidative, and bone-functional endpoints. Relevant measurements include TNF-α, IL-1β, IL-6, and IL-17; intracellular and mitochondrial ROS; antioxidant capacity; NLRP3 activation; cleaved caspase-1; mature IL-1β and IL-18; osteoclast resorption; and osteoblast-mediated matrix formation and mineralization. Controls should include unstimulated constructs, cytokine-only and ROS-induction controls, antioxidant or NLRP3-inhibition controls, hypoxia controls, and cytotoxicity controls. Because no universal cytokine or ROS threshold applies across cell sources and platforms, disease induction should be defined by a reproducible functional effect within a noncytotoxic range. Increased ROS or inflammasome markers without corresponding changes in bone formation or resorption are insufficient to establish osteoporosis-related inflammatory dysfunction.

### 2.4 Vascular dysfunction and impaired angiogenic-osteogenic coupling

Bone vasculature supports oxygen and nutrient delivery, metabolic exchange, and the trafficking of progenitor and immune cells within the bone marrow microenvironment (Ramasamy, 2017). Endothelial cells also regulate osteogenesis through paracrine signaling and their spatial association with osteoprogenitor cells. Type H vessels, characterized by high expression of CD31 and endomucin, are enriched near osteogenic regions and have been linked to the coupling of angiogenesis and bone formation (Peng et al., 2020). Experimental studies further show that this vascular subtype maintains perivascular osteoprogenitors and declines with age. Bone vascular function should therefore be considered an active regulator of remodeling rather than a passive nutrient-delivery system.

Aging, estrogen deficiency, chronic inflammation, and oxidative stress can disrupt endothelial survival, angiogenic signaling, and microvascular organization. Reduced vascular density or function may limit oxygen and nutrient delivery and weaken endothelial support for osteoblast differentiation. Conversely, inflammatory cytokines and ROS can impair endothelial integrity and increase vascular permeability. Disruption of angiogenic signaling has been implicated in osteoporosis pathogenesis, although the direction and magnitude of vascular changes may vary with disease subtype, skeletal site, and stage (James et al., 2024). Vascular dysfunction should therefore be defined by functional changes rather than by endothelial-cell abundance alone.

Vascular and bone dysfunction can form a reinforcing feedback loop. Reduced perfusion can increase local hypoxia, metabolic stress, and inflammatory signaling, thereby suppressing osteoblast function and promoting a pro-resorptive microenvironment. In turn, matrix degradation and inflammatory activation can alter endothelial behavior and further destabilize the vascular niche. These interactions are spatially dependent because endothelial support is strongest when vascular structures are positioned near osteogenic cells and remodeling surfaces. They are also time-dependent: early vascular impairment may precede defective bone formation, whereas prolonged hypoperfusion can produce nonspecific tissue injury.

These features have direct implications for model design. Two-dimensional endothelial culture remains appropriate for examining isolated angiogenic pathways or endothelial secreted factors. A three-dimensional platform is justified when the objective is to reproduce vascular network assembly, lumen formation, perfusion, endothelial permeability, oxygen gradients, or the spatial coupling of endothelial and osteogenic cells. Endothelial cells should be incorporated at a defined stage relative to matrix formation and osteogenic differentiation. Depending on the intended application, they may be distributed throughout the bone-like compartment, positioned in an adjacent vascular compartment, or organized around perfusable channels. The selected arrangement should distinguish direct cell-cell contact, paracrine signaling, and perfusion-mediated effects.

Validation should extend beyond endothelial-marker expression. CD31, endomucin, VE-cadherin, and von Willebrand factor can confirm endothelial identity, but they do not establish a functional vascular network. Morphologic endpoints should include network length, branch density, lumen formation, and spatial continuity. Functional endpoints should include perfusion, permeability, solute transport, and oxygen distribution, together with downstream effects on matrix formation, mineralization, and osteoclast activity. CD31 and endomucin coexpression alone should be described as a Type H-like phenotype unless vessel identity is supported by appropriate spatial, molecular, and functional evidence.

Controls should include constructs without endothelial cells, nonperfused vascularized constructs, perfused channels without endothelial lining, and defined proangiogenic or antiangiogenic interventions. Construct size and oxygen availability should also be controlled to distinguish disease-related vascular dysfunction from diffusion-limited hypoxia. A valid vascular phenotype should show a reproducible loss of network organization or transport function together with impaired bone formation or remodeling. Endothelial-marker changes without lumen formation, perfusion, or a corresponding bone-functional effect are insufficient to demonstrate osteoporosis-related vascular dysfunction.

### 2.5 Matrix alterations and dysregulated mechanical signaling

The extracellular matrix provides both structural support and mechanical signals that regulate bone-cell behavior. Its collagen organization, mineral content, porosity, degradability, and stiffness influence osteoblast differentiation, osteocyte maturation, osteoclast attachment, and the transmission of mechanical forces (Wang L. et al., 2022). In osteoporosis, deterioration of matrix quality and bone microarchitecture reduces mechanical competence and alters the local strain environment. Matrix abnormalities should therefore be considered part of the disease mechanism rather than only technical variables used to construct a three-dimensional model.

Osteocytes are the principal mechanosensory cells in bone. Mechanical strain and load-induced fluid flow are transmitted through the mineralized matrix and lacunar-canalicular network, where they activate integrin-, ion-channel-, cytoskeletal-, and Wnt-related signaling pathways (Qin et al., 2020). Physiological loading generally suppresses SOST/sclerostin expression and promotes Wnt/β-catenin-dependent bone formation. In contrast, mechanical unloading, immobilization, aging, or microgravity can increase sclerostin activity, suppress osteoblast function, and favor bone resorption (Chen et al., 2025). Changes in osteocyte-derived RANKL and OPG may further connect mechanical unloading to osteoclast activation.

Vascular and bone dysfunction can interact through reciprocal signaling. Reduced skeletal blood flow impairs endothelial Notch signaling, angiogenesis, and osteogenesis (Ramasamy et al., 2016). Hypoxia has been associated with reduced osteoblast differentiation and matrix mineralization and with increased osteoclast maturation and resorptive activity, although these effects vary with oxygen severity and exposure duration (Usategui-Martín et al., 2022). Conversely, bone-cell activity feeds back on the vascular compartment: preosteoclast-derived PDGF-BB promotes type H vessel formation, whereas an osteoporotic bone-on-a-chip microenvironment has been linked to increased vascular permeability (Lee et al., 2024). Endothelial aging can further suppress Wnt-dependent osteogenesis in mesenchymal stromal cells through paracrine miR-31-5p signaling (Fleischhacker et al., 2024). These interactions are spatially dependent because type H capillaries maintain closely associated perivascular osteoprogenitors. For model design, hypoperfusion and hypoxia should therefore be applied with defined intensity and duration so that early vascular signaling defects can be distinguished from nonspecific injury caused by prolonged oxygen and nutrient deprivation.

These relationships have direct implications for model design. Two-dimensional culture remains suitable for examining individual mechanosensors or the response of isolated cells to substrate stiffness. A three-dimensional platform is justified when the objective is to reproduce matrix-mediated load transfer, fluid flow around embedded osteocytes, spatial variation in mineralization, or mechanically regulated formation-resorption coupling. Osteocyte-like cells should be embedded within a matrix that permits force transmission and fluid exchange, while osteoblasts and osteoclasts should occupy matrix regions appropriate for formation and resorption. Static culture should not automatically be described as mechanical unloading unless it is compared with a defined physiological loading condition.

Matrix maturation should precede mechanical testing when the study addresses adult bone remodeling. Mechanical inputs may include cyclic compression, tensile strain, perfusion-induced shear stress, or controlled changes in matrix stiffness. The loading magnitude, frequency, duration, waveform, and number of cycles should be reported together with construct geometry and material properties. Acute mechanosensitive responses should be distinguished from longer-term changes in matrix formation, mineralization, or resorption.

Validation should confirm both delivery of the mechanical input and the resulting biological response. Engineering measurements should include matrix stiffness, mineral content, porosity, deformation, local strain, flow rate, or wall shear stress, as appropriate. Biological readouts may include SOST/sclerostin, Wnt/β-catenin activity, DMP1, PHEX, osteogenic differentiation, matrix deposition, mineralization, and osteoclast resorption. Controls should include unloaded or sham-loaded constructs, loading-dose controls, matrix-matched controls, and mechanosignaling-specific interventions. Where possible, pathway inhibition or anti-sclerostin treatment should be used to determine whether the functional response is mediated by the proposed mechanism.

No universal loading or matrix-stiffness threshold applies across platforms because the effective cellular stimulus depends on construct geometry, matrix composition, and measurement method. Each study should therefore define a noninjurious operating range using viability, structural integrity, and dose-response testing. A valid mechanical phenotype should show a reproducible relationship between a calibrated mechanical input, a mechanosensitive molecular response, and a bone-functional outcome. Changes caused by construct damage, altered diffusion, or generalized loss of viability should be classified as engineering failure rather than osteoporosis-related mechanodysfunction.

### 2.6 Integrated causal network and minimum model requirements

The mechanisms described above form an interconnected network. Osteocyte dysfunction, immune activation, oxidative inflammation, vascular impairment, and abnormal mechanical signaling converge on reduced bone formation, enhanced resorption, and defective repair. These processes also reinforce one another; for example, inflammation increases osteoclastogenesis, matrix degradation releases additional inflammatory signals, and vascular or mechanical dysfunction further impairs osteocyte-mediated remodeling (Torres et al., 2023; Wu et al., 2025).

An osteoporosis-related three-dimensional bone model need not reproduce the entire network. Its complexity should reflect the intended application and the minimum causal pathway required to answer the research question. At a minimum, the model should connect a defined disease-inducing condition to a mechanistic response and a measurable bone-functional outcome. Spatial organization should support the selected mechanism, such as matrix-embedded osteocytes, surface-accessible osteoclasts, immune-cell contact or migration, endothelial compartments adjacent to osteogenic regions, or controlled transmission of mechanical stimuli. Disease induction should occur only after establishment of an appropriate baseline tissue phenotype unless developmental impairment is the specific objective.

Model validity requires more than altered molecular markers (Candarlioglu et al., 2022). A successful model should maintain viability and structural integrity, exhibit reproducible changes in matrix formation, mineralization, three-dimensional resorption, vascular transport, or mechanosensitive function, and show at least partial rescue after mechanism-specific intervention. Phenotypes primarily caused by cytotoxicity, diffusion-limited hypoxia, excessive inflammatory stress, or matrix failure should be considered engineering failures rather than evidence of osteoporosis. Because universal quantitative thresholds are not yet available, acceptance criteria should be predefined relative to matched healthy, disease-induction, intervention, and toxicity controls. These requirements provide the mechanistic basis for the modular design framework presented in Section 3.

## 3 Design requirements and validation criteria for next-generation osteoporosis models

**FIGURE 2:**
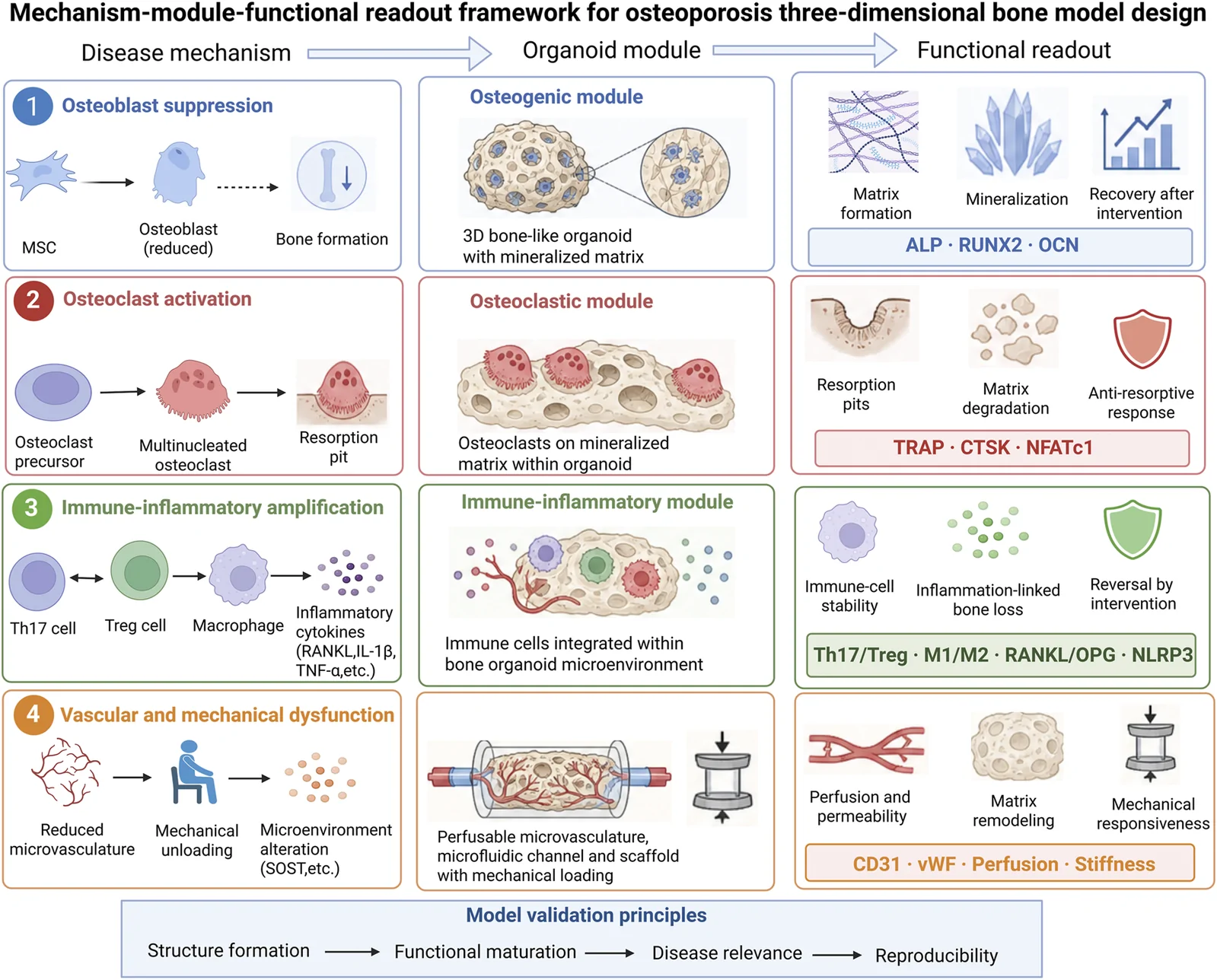
Mechanism–module–functional readout framework for osteoporosis-related three-dimensional bone model design. The framework maps representative osteoporosis mechanisms to selectable model modules and matched functional readouts. Osteoblast suppression, osteoclast activation, osteoimmune amplification, vascular dysfunction, and altered mechanical signaling are translated into osteogenic, osteoclastic, immune-inflammatory, vascular, and matrix–mechanical requirements, respectively. For visual clarity, the vascular and matrix–mechanical modules are presented together in the fourth row because perfusion, matrix remodeling, and mechanical responsiveness are functionally interconnected; however, they remain independently selectable modules and do not require simultaneous incorporation. The terms “organoid module” and “bone organoid” used within the figure serve as concise visual shorthand for the broader category of osteoporosis-related three-dimensional bone models, encompassing self-organizing bone organoids, engineered organoid-like bone models, and bone-on-a-chip systems. Module selection should be guided by the intended application, and not every model is expected to incorporate all components. Functional validation should progress from structural formation to functional maturation, disease relevance, and reproducibility. Created with BioRender.com. Publication license number: RJ2A50XLZV.

The mechanism-module-functional readout framework is proposed as a design scheme rather than a validated universal protocol (Figure 2). It links selected osteoporosis mechanisms to cellular or microenvironmental modules and predefined functional endpoints. Because evidence maturity differs among modules, the following sections distinguish established capabilities from partially validated and prospective features.

Model complexity should match the intended application, and modules should be included only when required by the selected causal pathway. Two-dimensional culture remains suitable for isolated signaling, lineage differentiation, and screening. A three-dimensional platform is justified when the research question depends on cell–matrix architecture, embedded osteocyte signaling, surface resorption, spatial gradients, multicellular interactions, perfusion, or mechanical force transmission. Each module is therefore evaluated by its minimum components, spatial and temporal organization, engineering inputs, functional endpoints, controls, validation criteria, and evidence level. A valid model should connect disease induction to a mechanistic response, a reproducible bone-functional phenotype, and mechanism-specific rescue.

### 3.1 Osteogenesis module: modeling impaired bone formation

The osteogenesis module should reproduce disease-related impairment of matrix production and mineralization. Three-dimensional osteoblast–osteocyte constructs subjected to estrogen withdrawal have reproduced changes in mineralization, mechanosensitivity, and pro-osteoclastogenic RANKL/OPG signaling (Naqvi et al., 2020). This study establish technical feasibility, but osteoporosis-specific calibration and cross-platform validation remain limited. The principal challenge is therefore not generating mineralized tissue, but distinguishing disease-specific osteogenic dysfunction from donor variability, matrix effects, or reduced cell viability.

Two-dimensional culture remains appropriate for studying isolated osteogenic pathways and lineage differentiation. A three-dimensional platform is justified when the research question requires cell-derived matrix deposition, spatially heterogeneous mineralization, long-term maturation, or osteocyte-related regulation (Finlay et al., 2023). The minimum configuration should include a defined osteoprogenitor source and a matrix with controlled stiffness, mineral content, porosity, and degradability. Osteocyte-like cells should be added only when the selected mechanism involves Wnt/sclerostin signaling or formation–resorption coupling (Marini et al., 2023).

Osteogenic dysfunction may be induced by estrogen withdrawal, inflammatory or oxidative stimulation, senescence, or Wnt inhibition (Cheng et al., 2022). Disease induction should follow establishment of a stable baseline unless impaired tissue development is the intended phenotype. Patient-derived cells may improve clinical relevance but require matched healthy donors or isogenic controls to separate disease effects from donor-specific variation.

Validation should combine lineage markers, including ALP, RUNX2, SP7, COL1A1, BGLAP, and SPP1 (Zainal Ariffin et al., 2022), with functional measurements of matrix deposition, calcium accumulation, mineralized volume, and three-dimensional structure. Wnt-related models should additionally assess β-catenin activity, SOST, and DKK1 (Cao and Wang, 2026). A valid phenotype should maintain viability, show a reproducible reduction in bone-forming function, and exhibit at least partial rescue after mechanism-specific intervention. Marker changes without reduced matrix formation or mineralization are insufficient, whereas phenotypes caused by cytotoxicity, poor matrix compatibility, or altered proliferation should be classified as model failure.

### 3.2 Osteoclast module: modeling excessive bone resorption

The osteoclast module should distinguish precursor differentiation, functional maturation, matrix resorption, and coupling to bone formation. M-CSF and RANKL can reliably induce osteoclast differentiation in two-dimensional monoculture or osteoblast–osteoclast coculture. A three-dimensional platform is therefore unnecessary when the objective is limited to RANKL signaling or lineage differentiation. Its added value lies in reproducing surface-localized resorption, sealing-zone formation, matrix-dependent maturation, and spatial interactions with osteoblasts or osteocytes.

Recent bone-on-a-chip platforms have enabled spatially confined, noninvasive analysis of human osteoclast resorption within biomimetic mineralized matrices (Conceição et al., 2026) and have integrated osteoclast-mediated resorption with osteoblast-driven matrix formation under perfused culture conditions (Stelzer et al., 2026). However, these studies primarily establish feasibility; standardized measurement of long-term three-dimensional resorption and subsequent bone formation remains limited. The main engineering challenges are therefore the combined effects of precursor source and maturation, matrix resorbability, access to the matrix surface, coculture-medium compatibility, and quantitative functional measurement.

The minimum configuration should include a defined monocyte–macrophage-lineage precursor and a matrix that supports adhesion, polarization, sealing-zone formation, and cell-mediated degradation (Veis and O'Brien, 2023). Osteoblasts, osteocytes, stromal cells, or immune cells should be added only when endogenous RANKL/OPG regulation or formation–resorption coupling is being investigated. Osteoclast precursors should be introduced after matrix maturation and positioned on an accessible mineralized surface. If coupling is examined, the resorption phase should precede assessment of osteoblast recruitment and matrix restoration (Remmers et al., 2020). High exogenous RANKL may confirm osteoclast competence but does not alone establish an osteoporosis-specific phenotype; disease relevance requires altered endogenous RANKL/OPG regulation, estrogen-deficient supporting cells, or a defined inflammatory stimulus.

TRAP-positive multinucleated cells and expression of ACP5, CTSK, NFATC1, and MMP9 indicate differentiation but not functional resorption. Validation should additionally assess F-actin rings, polarization, matrix acidification, and resorption area, depth, or volume using appropriate three-dimensional imaging. Controls should include precursor-free matrices, M-CSF-only conditions, matrix-matched controls, two-dimensional differentiation controls, and antiresorptive or pathway-specific interventions. A valid phenotype should maintain viability, show reproducibly increased matrix resorption, and respond to mechanism-specific inhibition. Marker changes without resorption indicate incomplete maturation, whereas passive matrix dissolution, cytotoxicity, or loss of matrix integrity should be classified as model failure.

### 3.3 Osteoimmune module: modeling inflammatory regulation of bone remodeling

The osteoimmune module should determine whether a defined immune perturbation alters bone formation or resorption. It is optional and should include only the immune population required by the research question. Current evidence for T-cell, B-cell, and macrophage regulation of bone remodeling derives mainly from patient studies, animal models, and conventional cocultures (Valverde and Naqvi, 2025); direct validation in osteoporosis-related organoids remains limited.

Two-dimensional or Transwell systems remain appropriate for testing individual cytokines, immune-cell products, and paracrine interactions. A three-dimensional platform is justified when the question depends on immune-cell migration, contact-dependent signaling, cytokine gradients, matrix retention of inflammatory mediators, or the spatial association of immune activation with bone formation and resorption. The principal engineering challenges are compatibility between immune and bone-cell media, maintenance of immune-cell viability and activation state, donor variability, and prevention of excessive inflammation that causes nonspecific tissue injury.

The minimum configuration should combine a mature bone-forming construct with one defined immune-cell population. Direct coculture is appropriate for contact-dependent mechanisms, whereas separate compartments should be used to isolate migration or paracrine signaling (He et al., 2024). Immune cells should generally be introduced after establishment of a stable bone phenotype. Disease induction may use defined cytokines, estrogen-deficient immune conditions, or controlled immune-cell activation. Lipopolysaccharide can model inflammatory stress but should not be considered osteoporosis-specific without a corresponding, noncytotoxic bone phenotype.

Validation should link immune state to bone function. Relevant measurements include the Th17/Treg balance, B-cell RANKL/OPG expression, macrophage activation profiles, inflammatory cytokines, and, where mechanistically relevant, ROS, mitochondrial ROS, NLRP3, caspase-1, and IL-18 (Dominic et al., 2022). These readouts should be accompanied by changes in matrix formation, mineralization, osteoclast differentiation, or three-dimensional resorption. Controls should include bone constructs without immune cells, unstimulated immune cells, cytokine-only conditions, viability controls, and mechanism-specific interventions. Cytokine elevation or altered immune-cell proportions without a reproducible bone-functional effect are insufficient, whereas generalized cell death, hypoxia, or loss of matrix integrity should be classified as model failure.

### 3.4 Vascular module: modeling vascular dysfunction and impaired angiogenic-osteogenic coupling

The vascular module should determine whether altered endothelial organization or transport affects bone formation and remodeling. It is most relevant to studies of vascular insufficiency, angiogenic–osteogenic coupling, oxygen delivery, or long-term culture of diffusion-limited constructs (Hann et al., 2021). Endothelial monoculture remains suitable for examining angiogenic signaling and secreted factors. A three-dimensional platform is justified when the question requires network assembly, lumen formation, perfusion, permeability, oxygen gradients, or spatial interactions between endothelial and osteogenic cells.

Scaffold-free bone organoids incorporating dental pulp stem cells have demonstrated vascular-like differentiation and sprouting (Li et al., 2023), while a humanized vascularized bone model has reproduced estrogen-withdrawal-associated changes in vessel-like structures and mineralization. These studies support the feasibility of vascularized bone models but do not establish universal criteria for mature or perfusable vasculature (Su et al., 2025). The main challenges remain endothelial-cell source, compatibility with osteogenic media, network stability, functional perfusion, and separation of disease-related vascular dysfunction from diffusion-limited hypoxia.

The minimum configuration should combine a defined endothelial population with an osteogenic construct. Perivascular cells should be added only when vessel stabilization or endothelial–stromal signaling is under investigation. Endothelial cells may be distributed within the osteogenic compartment, positioned adjacent to it, or organized around microfluidic channels. The arrangement should reflect whether the model examines direct contact, paracrine signaling, or perfusion-mediated transport. For adult osteoporosis modeling, vascular function should be established before estrogen withdrawal, inflammatory or oxidative stimulation, or controlled reduction of perfusion.

Validation should extend beyond CD31, endomucin, VE-cadherin, and von Willebrand factor expression. Morphologic assessment should include network length, branching, lumen formation, and spatial continuity, whereas functional assessment should measure perfusion, permeability, solute transport, and oxygen distribution. CD31 and endomucin coexpression should be described as a Type H-like phenotype unless supported by appropriate spatial and functional evidence (Zhao and Xie, 2020). Vascular outcomes should also be linked to matrix formation, mineralization, or osteoclast activity.

Controls should include constructs without endothelial cells, nonperfused vascularized constructs, perfused channels without endothelial lining, oxygen-matched controls, and vascular-targeted interventions. A valid phenotype should show reproducible loss of vascular organization or transport together with a bone-functional effect and at least partial rescue after mechanism-specific intervention. Endothelial-marker changes without functional transport are insufficient, whereas central necrosis, uncontrolled leakage, or network collapse caused by culture failure should not be interpreted as osteoporosis-related vascular dysfunction.

### 3.5 Matrix–mechanical module: modeling matrix deterioration and dysregulated mechanosignaling

The matrix–mechanical module should determine whether altered matrix properties or mechanical stimulation affect osteocyte signaling and bone remodeling. It is relevant when the research question involves matrix quality, mechanical unloading, fluid flow, or mechanoregulated formation–resorption coupling. Two-dimensional systems remain suitable for studying individual mechanosensors or substrate stiffness. A three-dimensional platform is justified when the response depends on matrix-mediated force transmission, embedded osteocytes, local fluid flow, spatially heterogeneous mineralization, or multicellular remodeling.

Matrix-inspired bioinks have supported the formation of large self-mineralizing bone organoids, while long-term cyclic loading of bioprinted mesenchymal stromal cell constructs has enhanced mineral density, stiffness, osteocyte differentiation, and lacunar–canalicular network formation (Zhang et al., 2022). These studies demonstrate engineering feasibility but primarily address tissue formation and regeneration rather than osteoporosis-specific mechanodysfunction. They also do not establish universal loading thresholds because the cellular stimulus depends on construct geometry, material properties, and loading method.

The minimum configuration should include osteogenic or osteocyte-like cells embedded in a matrix with defined stiffness, mineral content, porosity, and degradability. Osteoclasts should be added only when mechanically regulated resorption is investigated. Matrix maturation should precede loading when the objective is to model adult bone remodeling. Mechanical inputs may include cyclic compression, tensile strain, or perfusion-induced shear stress. Static culture should not be described as unloading unless it is compared with a defined loaded condition. Loading magnitude, frequency, duration, waveform, and cycle number should be reported together with construct geometry and matrix properties. Local deformation, strain, flow, or shear stress should be measured or estimated whenever possible.

Validation should confirm both delivery of the mechanical input and the resulting biological response. Engineering measurements should include matrix stiffness, mineral content, porosity, deformation, local strain, flow rate, or shear stress. Biological readouts may include SOST/sclerostin, Wnt/β-catenin activity, DMP1, PHEX, YAP/TAZ signaling, matrix deposition, mineralization, and osteoclast resorption. Controls should include unloaded or sham-loaded constructs, loading-dose controls, matrix-matched controls, and mechanosignaling-specific interventions. A valid phenotype should connect a calibrated mechanical input to a mechanosensitive response and a reproducible bone-functional outcome. Responses caused by construct damage, altered diffusion, or generalized loss of viability should be classified as engineering failure.

### 3.6 Module integration and hierarchical validation

Modules should be integrated incrementally rather than combined by default. A core model may reproduce impaired bone formation or excessive resorption, while a coupled model includes both processes. Immune, vascular, or mechanical components should be added only when required by the research question. Existing studies have demonstrated selected multicellular, vascularized, and localized remodeling models, but a universally transferable, plug-and-play platform has not been established.

Each added module should meet three criteria: compatibility, functional contribution, and baseline stability. The core construct should first establish predefined viability, matrix, and bone-functional benchmarks. After integration, the new component should retain its intended function without disrupting the baseline phenotype through incompatible media, altered diffusion, or nonspecific cellular stress. A platform should be considered modular only when a component can be added or removed without redesigning the entire system. If substantial reoptimization is required, matched controls are needed to separate module-specific effects from changes in culture conditions.

Validation should proceed hierarchically. Structural validation should confirm tissue integrity and cellular organization. Module-specific validation should demonstrate matrix formation, resorption, immune regulation, vascular transport, or mechanosensitive responses. Disease validation should link a defined induction condition to a reproducible functional phenotype, while mechanistic validation should show at least partial rescue after pathway-specific intervention. Reproducibility should be assessed across batches, donors, and, where feasible, laboratories (Wang et al., 2025b).

Composite functional endpoints should be prioritized over isolated marker changes. Drug-screening models additionally require stability, dose–response relationships, and scalability, whereas patient-derived models require association with relevant clinical features before predictive value can be claimed. Failure to maintain viability, structural integrity, or the core baseline phenotype after module addition should be classified as integration failure rather than increased disease complexity. The construction requirements, functional endpoints, validation criteria, and evidence maturity of each module are summarized in Tables 1, 2.

**TABLE 1:** Mechanism-informed construction requirements for osteoporosis-related three-dimensional bone model modules.

Module and pathological feature	Minimum biological components	Matrix and spatial organization	Disease induction and timing
Osteogenesis: impaired bone formation	Defined osteoprogenitors; osteocyte-like cells when Wnt/sclerostin signaling is investigated	Tunable mineralized matrix supporting cell embedding, matrix deposition, and spatially resolved mineralization	Apply estrogen withdrawal, inflammatory or oxidative stress, senescence, or Wnt inhibition after baseline matrix formation
Osteoclast: excessive resorption and remodeling uncoupling	Monocyte–macrophage-lineage precursors; osteoblasts or osteocytes when endogenous RANKL/OPG regulation is required	Resorbable mineralized surface accessible to osteoclasts; separate formation region when coupling is examined	Add precursors after matrix maturation; induce endogenous RANKL/OPG imbalance or controlled inflammation; assess formation after resorption
Osteoimmune: inflammatory amplification of bone loss	One defined immune population, such as T cells, B cells, or macrophages, combined with a mature bone construct	Direct contact for cell-dependent mechanisms or compartmentalization for migration and paracrine signaling	Introduce immune cells after establishment of the bone baseline; apply controlled immune activation or estrogen-deficient conditions
Vascular: impaired angiogenic–osteogenic coupling	Endothelial and osteogenic cells; perivascular cells only when vessel stabilization is required	Endothelial networks adjacent to or integrated with osteogenic regions; patent lumens and perfusable channels when transport is evaluated	Establish vascular function before estrogen withdrawal, inflammatory or oxidative stimulation, or controlled perfusion reduction
Matrix–mechanical: matrix deterioration and impaired mechanosignaling	Osteogenic or osteocyte-like cells; osteoclasts only when mechanically regulated resorption is examined	Matrix with defined stiffness, mineral content, porosity, degradability, and geometry capable of transmitting load or fluid flow	Apply calibrated compression, strain, or perfusion-induced shear after matrix maturation; report loading magnitude, frequency, duration, and waveform

Modules should be selected according to the intended application and need not be combined in every model.

**TABLE 2:** Functional validation criteria and evidence maturity of osteoporosis-related three-dimensional bone model modules.

Module	Primary functional endpoints	Essential controls and failure criteria	Current evidence maturity
Osteogenesis	Matrix deposition, mineralized volume, three-dimensional structure, Wnt/β-catenin activity, SOST, and DKK1	Include healthy, vehicle, viability, and acellular matrix controls. Require reduced bone-forming function and mechanism-specific. Marker changes alone are insufficient	Three-dimensional osteogenesis is established, but osteoporosis-specific calibration remains limited
Osteoclast	Polarization, sealing-zone formation, matrix acidification, and resorption area, depth, or volume	Include M-CSF-only, precursor-free, and matrix-matched controls. Exclude passive matrix degradation and cytotoxicity. Require antiresorptive or pathway-specific rescue	Two-dimensional differentiation is established; localized three-dimensional resorption is partially validated, whereas sequential remodeling coupling remains underdeveloped
Osteoimmune	Immune-cell state, RANKL/OPG balance, cytokine production, osteoclast resorption, matrix formation, and mineralization	Include bone-only, unstimulated immune-cell, cytokine-only, and viability controls. Immune changes without a bone-functional effect are insufficient	Osteoimmune mechanisms are well supported, but functional reconstruction in osteoporosis-related three-dimensional bone models remains largely prospective
Vascular	Network continuity, lumen formation, perfusion, permeability, oxygen distribution, and associated bone-functional changes	Include nonvascularized, nonperfused, channel-only, and oxygen-matched controls. Endothelial markers without functional transport are insufficient	Vascular-like organization is feasible, but mature perfusion and osteoporosis-specific vascular dysfunction remain incompletely validated
Matrix–mechanical	Local deformation or shear stress, SOST, Wnt/β-catenin and YAP/TAZ activity, matrix formation, mineralization, stiffness, and resorption	Include sham-loaded, loading-dose, and matrix-matched controls. Exclude construct damage, altered diffusion, and generalized viability loss	Mechanical loading is technically feasible, but physiological calibration and osteoporosis-specific validation remain prospective

A valid osteoporosis phenotype requires preserved viability and structural integrity, a mechanism-consistent response, a corresponding functional change, and at least partial rescue after mechanism-specific intervention. When universal thresholds are unavailable, acceptance criteria should be predefined relative to matched controls.

## 4 Construction strategies and platform selection for modular osteoporosis models

The modules defined in Section 3 must be implemented through coordinated selection of cell source, matrix, and construction platform. No single method is optimal for all applications. Self-organizing culture, scaffold-based systems, bioprinting, and microfluidic platforms provide different levels of spatial control, matrix tunability, perfusion, mechanical stimulation, longitudinal assessment, throughput, and reproducibility. Platform selection should therefore be guided by the intended mechanism and functional endpoint rather than by technical complexity alone.

Modularity should be evaluated experimentally. Immune, vascular, or mechanical components should be added without substantially altering the viability, structure, or baseline function of the core construct. If integration requires changes in medium, matrix, geometry, or culture duration, matched controls are needed to distinguish module-specific effects from platform reoptimization. The following sections define the boundaries of bone organoid models, examine how cells and matrices interact with construction strategies, and compare platform suitability for modular expansion and functional validation.

### 4.1 Conceptual boundaries and classification of bone organoid models

Organoids are generally defined as three-dimensional tissue models derived from stem, progenitor or tissue-resident cells that reproduce selected structural and functional properties of the corresponding tissue through self-organization and lineage-specific differentiation (Zhao et al., 2022). In bone research, however, the term “bone organoid” is also frequently applied to engineered constructs produced through scaffold-based culture, multicellular assembly, bioprinting or microfluidic technologies. Because these approaches differ substantially in their capacity for self-organization, spatial patterning and functional maturation, not every three-dimensional bone culture should be classified as a bone organoid. The terminology used to describe a model should reflect its biological properties and construction principles rather than its three-dimensional morphology alone.

Bone-related three-dimensional models can be broadly classified into four categories. First, self-organizing bone organoids are derived from stem or progenitor cells and exhibit intrinsic cellular organization, lineage differentiation and the formation of bone-like extracellular matrix. Second, engineered organoid-like bone models combine selected bone-associated cell populations with biomimetic matrices, defined spatial assembly or bioprinting to reproduce specific structural or functional features of bone tissue. Third, bone-marrow organoids place greater emphasis on interactions among stromal, hematopoietic, immune and endothelial components and are therefore particularly relevant to the investigation of bone-marrow niches and osteoimmune regulation (Scala et al., 2026). Fourth, bone-on-a-chip and related microphysiological systems use microfluidic compartmentalization, perfusion or controlled mechanical stimulation to model dynamic cell–cell and cell–matrix interactions. These systems may incorporate organoid components but should not automatically be considered organoids.

Osteochondral organoids and osteochondral-on-chip models constitute a related but distinct category. They are primarily designed to reproduce the bone–cartilage interface, endochondral ossification, joint degeneration or osteochondral repair (Fu et al., 2025). Although they can provide insights into bone formation and remodeling, their relevance to osteoporosis depends on whether they reproduce disease-specific processes such as impaired osteogenesis, enhanced resorption, osteoimmune dysregulation, vascular insufficiency or altered mechanical responses. Similarly, bioprinted or scaffold-based bone constructs may offer precise control over geometry, matrix composition and cellular distribution, but their classification as organoids requires evidence of sustained multicellular organization and tissue-relevant function rather than fabrication complexity alone (Gao et al., 2025).

In this review, the term osteoporosis-related three-dimensional bone models refers primarily to a human-relevant three-dimensional model that reproduces one or more defined aspects of osteoporotic bone biology through organized multicellular and matrix interactions. Relevant functions may include osteoid formation, mineralization, osteoclast-mediated resorption, osteoimmune regulation, vascular support or mechanosensitive signaling. Models that lack clear evidence of self-organization but reproduce these processes through controlled engineering are described more cautiously as organoid-like bone models or bone microphysiological systems. Regardless of terminology, each model should be evaluated according to its cellular composition, construction method, degree of spatial organization, functional maturity, disease relevance and reproducibility.

### 4.2 Co-selection of cell sources, matrices, and construction platforms

Cell source and matrix should be selected together with the construction platform because each method imposes different requirements for cell aggregation, encapsulation, spatial patterning, perfusion, and mechanical stimulation. Cell selection should therefore reflect both the disease mechanism and the technical environment rather than osteogenic potential alone (Kong et al., 2024).

Self-organizing systems are most compatible with expandable progenitor populations capable of aggregation, lineage differentiation, and matrix production. Primary or patient-derived cells may preserve disease-associated features but introduce donor variability and limited expansion. Induced pluripotent stem cells provide scalable and potentially isogenic cell sources, although incomplete maturation and batch variability remain important limitations. Scaffold-based and bioprinted models permit the defined incorporation of osteogenic, osteoclastic, immune, and endothelial populations,but encapsulated cells must tolerate material cross-linking, printing stress, and prolonged culture. Microfluidic platforms require cell numbers, adhesion properties, and media formulations compatible with compartmentalized culture and continuous flow.

Matrix selection should likewise follow platform function. Self-organizing models use minimal exogenous matrix and preserve cell-driven organization, but provide limited control over stiffness and mineral content. Scaffold-based systems allow collagen, GelMA, decellularized matrix, hydroxyapatite, or calcium phosphate composites to be selected according to adhesion, degradability, porosity, and mechanical requirements (Wang et al., 2024; Emami et al., 2025; Oh et al., 2025).

Additional cell populations should be incorporated only when they contribute a defined function. Osteoclast precursors require access to a resorbable surface, immune cells require compatible activation and migration conditions, and endothelial cells require space for network and lumen formation. Before integration, the core construct should meet predefined viability, structural, and bone-functional benchmarks. After integration, changes in medium, matrix, cell ratio, oxygenation, and culture duration should be documented. If these changes alter the baseline phenotype, a matched reoptimized core control is required to separate module-specific effects from platform effects.

### 4.3 Comparative performance and modular extensibility of construction strategies

Self-organizing, scaffold-based, bioprinted, and microfluidic systems provide different combinations of biological organization and engineering control. Their suitability should be evaluated according to spatial control, matrix tunability, perfusion, mechanical stimulation, longitudinal assessment, throughput, reproducibility, and modular extensibility rather than technical sophistication alone (Zhong et al., 2024; Rayat Pisheh and Vaez, 2026). Self-organizing culture preserves intrinsic cell–cell interactions and developmental-like matrix production but offers limited control over geometry, cellular position, and matrix properties. It can support moderate-to-high throughput when combined with microwells or automated aggregation, although size heterogeneity, diffusion limitations, and batch variability remain common (Zhao et al., 2024). Immune or endothelial cells can be introduced during aggregation, but later addition may disrupt tissue organization or produce uneven cell distribution.

Scaffold-based culture provides greater control over stiffness, porosity, mineral content, and degradation than self-organizing systems. It supports mechanical testing and sequential cell seeding, but perfusion depends on pore interconnectivity or integration with a bioreactor. Standardized materials can improve reproducibility, whereas natural matrices and decellularized tissues may introduce batch variation. Late addition of immune or vascular cells is feasible when the scaffold remains accessible, although embedded cells may require reconstruction of the original matrix.

Bioprinting offers high spatial control over cell distribution, matrix composition, compartment geometry, and mineral gradients. It can generate predefined osteogenic, resorptive, and vascular regions and facilitate registered longitudinal imaging. However, printability requirements may compromise matrix biology, and shear stress, cross-linking, and printing duration can reduce cell viability or alter function. Printed geometry is reproducible, but adding a new module often requires modification of the bioink, printing sequence, or construct design and may therefore represent platform reengineering rather than simple modular extension.

Microfluidic systems provide the strongest control over perfusion, solute gradients, shear stress, compartmentalized coculture, and repeated sampling (Kim et al., 2023; Salehi et al., 2024). They are well suited to longitudinal imaging and analysis of migration, paracrine signaling, vascular transport, or mechanically induced responses. Their limitations include fabrication and operational complexity, low tissue volume, material-dependent drug adsorption, and limited cross-platform standardization. Modular compartments can permit immune, vascular, or mechanical inputs to be added without reconstructing the core tissue, provided that ports, channels, and compatible media are incorporated during initial design.

Hybrid platforms can combine these advantages, for example, by embedding self-organized tissues within tunable scaffolds, bioprinting predefined compartments, or connecting constructs to microfluidic perfusion (Zhang et al., 2020; Strobel et al., 2023; Papp et al., 2024; Rasouli and Safari, 2025).

However, each added component should pass three tests: preservation of the core baseline phenotype, demonstration of its intended function, and removal or inhibition of the component without nonspecific construct failure. If immune, vascular, or mechanical integration requires changes in matrix, medium, geometry, or maturation state that alter the baseline, the resulting system should be treated as a new configuration and validated against a matched core control. The comparative performance and modular extensibility of these construction strategies are summarized in Table 3.

**TABLE 3:** Comparative characteristics of construction strategies for modular osteoporosis models.

Evaluation criterion	Self-organizing culture	Scaffold-based culture	Three-dimensional bioprinting	Microfluidic systems
Spatial control	L–M	M–H	H	H
Matrix tunability	L	H	H	M
Perfusion capacity	L	M	M	H
Mechanical stimulation	L	M–H	M–H	H
Longitudinal assessment	M	M	H	H
Throughput	M–H	M	L–M	M
Reproducibility	L–M	M–H	M–H	M
Modular extensibility	L–M	M	M	H
Principal advantage	Intrinsic cellular organization	Tunable matrix properties	Precise spatial patterning	Dynamic control and repeated sampling
Principal limitation	Heterogeneity and diffusion	Matrix-dependent variability	Printing stress and reengineering	Operational complexity and limited tissue volume

H, high; M, moderate; L, low. Ratings are qualitative and application-dependent. Modular extensibility indicates whether immune, vascular, or mechanical components can be incorporated without redesigning the core platform or altering its baseline phenotype.

### 4.4 Establishing fit-for-purpose quality control and provisional quantitative acceptance criteria

Quality control should be defined prospectively and matched to the intended application. Current organoid and microphysiological-system guidance does not support universal acceptance thresholds for matrix stiffness, mineralization, perfusion, vascular function, or bone resorption. Studies should therefore distinguish established standards from provisional, fit-for-purpose criteria derived from published guidance, historical controls, or expert consensus (Pamies et al., 2024).

Input-cell quality control should document cell identity, donor characteristics, tissue source, passage number, disease background, differentiation efficiency, and contamination status. Cultures should be mycoplasma-negative before model construction. Human pluripotent stem cell-derived models should additionally document cell-line authentication, pluripotency, genomic integrity, and consistency of directed differentiation (Ludwig et al., 2023). For osteoclast, immune, and endothelial components, cell purity, activation state, incorporation ratio, and timing of addition should be reported.

Material and fabrication parameters should include material lot, composition, cross-linking conditions, stiffness, porosity, mineral content, printing pressure and speed, nozzle diameter, perfusion rate, and culture duration (Malekpour and Chen, 2022; Xu et al., 2022). For bioprinted constructs, we recommend evaluating sterility, cytocompatibility, printability, structural fidelity, and post-printing cell viability as core process-quality attributes.

As a provisional process-acceptance criterion, post-fabrication viability should be at least 70% of that measured in a matched unprocessed or cast control. This threshold is adapted from ISO 10993–5, in which a viability reduction greater than 30% indicates cytotoxicity (Gruber and Nickel, 2023). Constructs below this threshold should be excluded or reoptimized. However, this value represents a minimum cytocompatibility criterion rather than evidence of functional maturity. The assay method, measurement time point, reference condition, and spatial sampling strategy should therefore be reported, and more stringent platform-specific thresholds should be established when sufficient historical data are available.

## 5 Evidence framework for three-dimensional bone model–enabled biomarker translation in osteoporosis

Not every molecule measured in an osteoporosis-related three-dimensional bone model is a biomarker. To avoid conflating mechanistic readouts with clinically validated tools, evidence should be classified into five categories: established clinical biomarkers, patient-associated candidate biomarkers, model-associated molecular signals, model-informed candidates subsequently confirmed in clinical samples, and biomarkers with demonstrated clinical predictive value. These terms represent different levels of evidence and should not be used interchangeably. Most current applications of osteoporosis-related three-dimensional bone models remain at the stage of mechanistic signal detection and candidate prioritization; evidence for subsequent clinical validation is limited, and prospective predictive utility has not been established.

### 5.1 Established clinical biomarkers and patient-associated candidates

Serum PINP and plasma β-CTX-I are established reference markers of bone formation and resorption, respectively (Vasikaran et al., 2023). They are used primarily to assess bone turnover, treatment response, and adherence. However, they provide limited information about the cellular source or local mechanism of bone loss and are affected by preanalytical, biological, renal, and assay-related variation. Bone mineral density and fracture outcomes should be treated as clinical comparators and outcomes rather than molecular biomarkers.

Patient studies have identified transcriptomic and single-cell signatures (Wang H. et al., 2025), circulating extracellular-vesicle miRNAs (Shi et al., 2022), and metabolite profilesassociated with osteoporosis (Wang et al., 2019). Multi-omics analyses may further identify molecular subtypes and candidate panels (Yuan et al., 2024; Wang et al., 2025c). These findings should be described as patient-associated candidates unless they have undergone analytical validation, independent cohort replication, adjustment for clinical confounders, and comparison with established clinical measures. Statistical association or computational feature selection alone does not demonstrate diagnostic or predictive utility.

### 5.2 Model-associated signals and their added value

Molecular changes measured only within an osteoporosis-related three-dimensional bone model—including SOST, the RANKL/OPG ratio, inflammatory cytokines, ROS-related signals, NLRP3 activation, cell-state signatures, or secreted metabolites—are mechanistic readouts rather than clinical biomarkers. Their value lies in determining whether a signal changes with a controlled disease perturbation, corresponds to impaired matrix formation or increased resorption, and is reversed by mechanism-specific intervention. Existing bone models can provide this functional context through estrogen withdrawal, inflammatory stimulation, multicellular remodeling, or vascularized culture (Park et al., 2021; Bukhari et al., 2025).

Direct patient-sample analysis captures systemic disease biology and clinical outcomes but is often observational, temporally limited, and influenced by age, medication, renal function, comorbidities, and tissue accessibility (Bhattoa et al., 2025). These three-dimensional bone models provide complementary information by enabling controlled perturbation, repeated sampling, cell-specific analysis, spatial mapping, and direct linkage of molecular signals to bone-functional outcomes. They may therefore help identify the cellular origin of a candidate and prioritize signals with mechanistic relevance.

This advantage has limits. These three-dimensional bone models do not reproduce the complete endocrine, renal, neural, metabolic, and pharmacokinetic environment of the patient. Signals generated by hypoxia, culture stress, matrix degradation, or immature cell states may have little clinical relevance (Yao et al., 2024). These three-dimensional bone models should therefore complement, rather than replace, direct analysis of patient serum, plasma, urine, bone marrow, or bone tissue.

### 5.3 From a model-associated signal to a clinically validated biomarker

Based on general biomarker-development principles, a defensible translation pathway can be organized into five stages (Ou et al., 2021). First, the candidate should be reproducibly associated with a defined model perturbation and bone-functional phenotype. Second, analytical and biological reproducibility should be established across batches, donors, and relevant platforms. Third, the signal should be detectable in an accessible clinical specimen and confirmed in appropriately matched patient samples. Fourth, its association with disease severity or treatment response should be replicated in an independent cohort and compared with PINP, β-CTX-I, bone mineral density, and established clinical risk factors. Fifth, prospective studies should determine whether the candidate improves prediction of fracture, treatment response, or adverse outcomes.

For the purposes of this review, a signal measured only in a three-dimensional bone model is a model-associated readout. After confirmation in patient samples, it may be described as a clinically supported candidate. Clinical predictive value should be claimed only when a pretreatment measurement predicts a subsequent outcome in an independent or prospective cohort and adds information beyond established predictors. A molecular change observed after drug exposure is a pharmacodynamic response marker, not necessarily a predictive biomarker.

Candidates should be excluded when their apparent association is explained by reduced viability, diffusion-limited hypoxia, matrix interference, generalized inflammation, or donor imbalance (Ahammed and Kalangi, 2024). Analytical sensitivity, specificity, assay stability, reference intervals, and clinical feasibility are also required before translation can be considered.

### 5.4 Drug-response biomarkers and patient stratification: Current boundaries

Patient-derived three-dimensional bone models may help prioritize candidate response markers by comparing osteogenic recovery, suppression of resorption, resolution of inflammation, or restoration of vascular support after treatment. However, an *in vitro* responder–nonresponder difference does not demonstrate clinical prediction. Such differences may arise from donor background, cell source, culture conditions, matrix properties, incomplete maturation, or differences in drug exposure.

Validation requires paired data from patient-derived three-dimensional bone models and the corresponding well-characterized patients treated with the same intervention. Pretreatment model-derived phenotypes and candidate measurements should be tested against subsequent changes in bone turnover markers, bone mineral density, fracture outcomes, adherence, and adverse events (Bhattoa et al., 2025). Assay reproducibility, construction time, cost, and the proportion of failed patient-derived cultures must also be reported before feasibility for stratification can be assessed.

At present, osteoporosis-related three-dimensional bone models should be viewed as platforms for mechanistic interpretation and candidate prioritization rather than as clinically validated tools for biomarker-guided prescribing. Their near-term contribution is to reduce large molecular candidate lists to smaller sets supported by causal and functional evidence. Translation to diagnostic, prognostic, or predictive use remains prospective and requires independent clinical validation.

## 6 Current limitations, failure criteria, and translational priorities

Osteoporosis-related three-dimensional bone models remain experimental platforms rather than standardized disease-modeling or clinical-prediction tools. Their translational value depends on whether compatible and functionally mature modules can generate reproducible, mechanism-specific phenotypes. Current limitations include cell and medium incompatibility, incomplete maturation, diffusion constraints, limited vascular function, insufficient mechanical calibration, inadequate three-dimensional measurements, donor and matrix variability, limited scalability, and weak clinical benchmarking (Xu et al., 2025).

### 6.1 Construction compatibility and functional maturity

Multicellular integration is constrained by differences in medium composition, oxygen demand, growth-factor dependence, and differentiation timeline. Conditions supporting osteogenesis may impair osteoclast, immune, or endothelial function, producing phenotypes that reflect culture incompatibility rather than osteoporosis biology (Sieberath et al., 2020). Sequential differentiation, compartmentalized culture, or controlled medium exchange may reduce these conflicts. Any resulting change in culture conditions should be accompanied by a matched core-construct control.

Cellular maturity should be established through function rather than marker expression alone. Osteoblast-lineage cells should produce organized mineralized matrix; osteocyte-like cells should exhibit matrix embedding, dendritic connectivity, and mechanosensitive signaling; osteoclasts should polarize and resorb mineralized matrix; and endothelial cells should form patent, perfusable networks. Vascular-like structures should not be considered functional vasculature without evidence of lumen formation, perfusion, barrier properties, and flow responsiveness.

Diffusion becomes increasingly restrictive as constructs enlarge and mineralize. Oxygen and nutrient gradients may induce central hypoxia, necrosis, oxidative stress, and inflammatory signaling that mimic osteoporosis-associated changes. Viability and oxygenation should therefore be evaluated spatially, with predefined limits for construct size and cell density. Similarly, mechanical stimulation should be calibrated according to the local strain experienced by the construct rather than device input alone. Loading that causes matrix damage, widespread cell death, or nonspecific stress responses should not be interpreted as physiological mechanotransduction.

### 6.2 Functional measurement, variability, and reproducibility

True three-dimensional bone resorption remains difficult to quantify. TRAP expression, osteoclast number, soluble degradation products, and two-dimensional resorption pits are informative but do not independently demonstrate volumetric matrix loss. Validation should combine osteoclast polarization with quantitative measurements of resorption volume, depth, surface area, or connectivity using three-dimensional imaging or paired structural analysis (Park et al., 2021). Results should be normalized to viable osteoclast number and available mineralized surface, with acellular controls used to exclude passive matrix degradation.

Most current assays are destructive and provide only terminal measurements. Repeated sampling, non-destructive imaging, stable reporters, or serial structural measurements are needed to determine whether resorption is followed by compensatory formation or persistent remodeling uncoupling. A single endpoint cannot establish the temporal sequence of an osteoporosis phenotype.

Primary and patient-derived cells introduce variability related to donor age, sex, disease severity, medication, passage number, and cryopreservation. Natural and decellularized matrices add variation in stiffness, mineral content, degradability, and retained bioactive factors. Studies should therefore include multiple donors, donor-matched controls, technical replicates, matrix-batch characterization, and predefined inclusion criteria. Highly osteoinductive matrices may mask impaired osteogenesis, whereas uncontrolled matrix degradation may be mistaken for osteoclast activity.

Scalability should be evaluated through usable construct yield, batch failure rate, variability, assay duration, cost, and compatibility with automated analysis. Increasing complexity generally reduces throughput and reproducibility; model complexity should therefore be matched to the intended application. Cross-laboratory studies should report cell source, medium composition, matrix properties, construct geometry, oxygenation, perfusion, loading parameters, culture timeline, and assay normalization. Shared protocols, reference materials, blinded sample exchange, and multicenter validation will be required to establish platform reproducibility (Jensen and Little, 2023).

### 6.3 Failure criteria and clinical translation boundaries

A valid osteoporosis phenotype should meet four requirements: preserved viability and structural integrity; a mechanism-consistent molecular response; a corresponding functional change in formation, resorption, vascular support, or mechanosensitivity; and at least partial rescue by a mechanism-specific intervention. Molecular changes without functional consequences are insufficient.

A model should fail disease validation when the phenotype is attributable to generalized toxicity, excessive hypoxia, nonspecific inflammatory stress, passive matrix degradation, incompatible media, or loss of a major cell population. Other failure conditions include absence of a functional endpoint, failure to reproduce the phenotype across independent batches, inability to achieve mechanism-specific rescue, and disruption of the baseline construct after adding a new module. Because universal thresholds are unavailable, acceptance criteria for viability, hypoxia, batch variation, and functional change should be defined prospectively relative to matched controls. Appropriate controls include untreated or healthy constructs, vehicle controls, toxicity or hypoxia controls, acellular matrices, and reoptimized core controls when module integration alters culture conditions.

Clinical benchmarking should compare model-derived phenotypes with bone mineral density, PINP, β-CTX, imaging characteristics, fracture history, and longitudinal treatment response (Cosman et al., 2024). Postmenopausal and inflammatory bone loss are currently more tractable because estrogen withdrawal, cytokine exposure, and RANKL/OPG dysregulation can be induced under controlled conditions. Glucocorticoid-induced and aging-related osteoporosis remain more difficult because their systemic endocrine, metabolic, muscular, renal, and long-term aging components are incompletely reproduced. Antiresorptive agents are currently more suitable for evaluation in osteoporosis-related three-dimensional bone models because their effects can be directly quantified through osteoclast differentiation and matrix resorption. In contrast, anabolic agents remain less readily modeled because their evaluation requires mature osteoblast–osteocyte signaling, physiologically calibrated mechanical loading, matrix maturation, and appropriate exposure kinetics.

## 7 Conclusion

This review proposes a mechanism–module–functional readout framework for the development and evaluation of osteoporosis-related three-dimensional bone models. The framework translates impaired osteogenesis–resorption coupling, osteoimmune dysregulation, vascular insufficiency and altered mechanical signaling into selectable model modules with predefined structural and functional endpoints. At present, the most realistic value of these models lies in providing controllable, human-relevant three-dimensional platforms for investigating multicellular interactions, linking molecular changes to functional bone phenotypes, evaluating early drug responses and prioritizing candidate biomarkers for subsequent validation. Across all categories—from self-organizing organoids to engineered constructs and bone-on-a-chip systems—these three-dimensional bone models should therefore be regarded as complementary intermediate platforms rather than substitutes for animal studies or clinical investigation. Their progression toward translational application requires stronger evidence of functional maturation, reproducibility across cell sources and experimental batches, and consistent performance across laboratories. Model-derived molecular and functional readouts must also be correlated with clinically relevant measures, including bone mineral density, bone-turnover markers, imaging characteristics, fracture risk and longitudinal treatment response. Prospective patient cohorts and independent validation studies will be essential to determine whether these models provide predictive information beyond established clinical indicators. Only through this stepwise evidence chain—from reproducible model phenotypes to prospective clinical correlation—could osteoporosis-related three-dimensional bone models support future biomarker translation, patient-stratification research and treatment-response assessment.
